# Supplementary material for: Ancient viral genomes reveal introduction of human pathogenic viruses into Mexico during the transatlantic slave trade
Source: eLife. 2021 Aug 5;10:e68612. doi: 10.7554/eLife.68612 (PMC8423449; doi:10.7554/eLife.68612)
Supplement: Transparent reporting form [file elife-68612-transrepform1.docx]

***eLife’s* transparent reporting form**

We encourage authors to provide detailed information *within their submission* to facilitate the interpretation and replication of experiments. Authors can upload supporting documentation to indicate the use of appropriate reporting guidelines for health-related research (see [EQUATOR Network](http://www.equator-network.org/%20)), life science research (see the [BioSharing Information Resource](https://biosharing.org/)), or the [ARRIVE guidelines](http://www.plosbiology.org/article/info:doi/10.1371/journal.pbio.1000412) for reporting work involving animal research. Where applicable, authors should refer to any relevant reporting standards documents in this form.

If you have any questions, please consult our Journal Policies and/or contact us: [editorial@elifesciences.org](mailto:editorial@elifesciences.org).

**Sample-size estimation**

- You should state whether an appropriate sample size was computed when the study was being designed
- You should state the statistical method of sample size computation and any required assumptions
- If no explicit power analysis was used, you should describe how you decided what sample (replicate) size (number) to use

Please outline where this information can be found within the submission (e.g., sections or figure legends), or explain why this information doesn’t apply to your submission:

Two skeletal collections were used for this study, the 21 Hospital San Jose de los Naturales (HSJN) samples were selected based on previous studies that described these individuals as African descendants. While the 5 samples from La Concepcion (COY) were chosen based on their conservation state, this information is mentioned in Methods (Sample selection and DNA extraction).

**Replicates**

- You should report how often each experiment was performed
- You should include a definition of biological versus technical replication
- The data obtained should be provided and sufficient information should be provided to indicate the number of independent biological and/or technical replicates
- If you encountered any outliers, you should describe how these were handled
- Criteria for exclusion/inclusion of data should be clearly stated
- High-throughput sequence data should be uploaded before submission, with a private link for reviewers provided (these are available from both GEO and ArrayExpress)

Please outline where this information can be found within the submission (e.g., sections or figure legends), or explain why this information doesn’t apply to your submission:

Due to the destructive process of samples, only one dental piece was used to generate the NGS libraries for each individual. Two in-solution captures were used to recover B19V and one for HBV (technical replicates), this information is mention in Methods (Capture-enrichment Assay).

Reconstructed genomes from this study are available in Genbank under accession number MT108214, MT108215, MT108216, MT108217. NGS reads used to reconstruct ancient viral genomes reported in this study are available in Dryad (<https://doi.org/10.5061/dryad.5x69p8d2s>).

The NGS data can be accessed through this private link (for reviewers) (<https://datadryad.org/stash/share/D9RPDa1FBj1sT5ry9M2Kchnn98VlCrPeVKaxzBQX974>).

**Statistical reporting**

- Statistical analysis methods should be described and justified
- Raw data should be presented in figures whenever informative to do so (typically when N per group is less than 10)
- For each experiment, you should identify the statistical tests used, exact values of N, definitions of center, methods of multiple test correction, and dispersion and precision measures (e.g., mean, median, SD, SEM, confidence intervals; and, for the major substantive results, a measure of effect size (e.g., Pearson's r, Cohen's d)
- Report exact p-values wherever possible alongside the summary statistics and 95% confidence intervals. These should be reported for all key questions and not only when the p-value is less than 0.05.

Please outline where this information can be found within the submission (e.g., sections or figure legends), or explain why this information doesn’t apply to your submission:

Description of the statistical methods used for Phylogenetic and isotope analyses can be found in methods and in the legends of Figure 3 and Figure 3-figure supplement 5-9 and Figure 4-figure supplements 1.3.

(For large datasets, or papers with a very large number of statistical tests, you may upload a single table file with tests, Ns, etc., with reference to sections in the manuscript.)

**Group allocation**

- Indicate how samples were allocated into experimental groups (in the case of clinical studies, please specify allocation to treatment method); if randomization was used, please also state if restricted randomization was applied
- Indicate if masking was used during group allocation, data collection and/or data analysis

Please outline where this information can be found within the submission (e.g., sections or figure legends), or explain why this information doesn’t apply to your submission:

Not applicable

**Additional data files (“source data”)**

- We encourage you to upload relevant additional data files, such as numerical data that are represented as a graph in a figure, or as a summary table
- Where provided, these should be in the most useful format, and they can be uploaded as “Source data” files linked to a main figure or table
- Include model definition files including the full list of parameters used
- Include code used for data analysis (e.g., R, MatLab)
- Avoid stating that data files are “available upon request”

Please indicate the figures or tables for which source data files have been provided:

Source data 1. Multi-fasta alignment of all genomes used to perform phylogenetic analysis of B19V.

Source data 2. Multi-fasta alignment of all genomes used to perform phylogenetic analysis of HBV.
